# Critical Quantum Metrology in the Non-Linear Quantum Rabi Model

**DOI:** 10.3390/e24081015

**Published:** 2022-07-22

**Authors:** Zu-Jian Ying, Simone Felicetti, Gang Liu, Daniel Braak

**Affiliations:** 1 School of Physical Science and Technology, Lanzhou University, Lanzhou 730000, China; 2 Institute for Complex Systems, National Research Council (ISC-CNR), 00185 Rome, Italy; 3 EP VI and Center for Electronic Correlations and Magnetism, University of Augsburg, 86135 Augsburg, Germany

**Keywords:** finite component phase transition, quantum metrology, quantum Rabi model, nonlinear coupling

## Abstract

The quantum Rabi model (QRM) with linear coupling between light mode and qubit exhibits the analog of a second-order phase transition for vanishing mode frequency which allows for criticality-enhanced quantum metrology in a few-body system. We show that the QRM including a nonlinear coupling term exhibits much higher measurement precisions due to its first-order-like phase transition at *finite* frequency, avoiding the detrimental slowing-down effect close to the critical point of the linear QRM. When a bias term is added to the Hamiltonian, the system can be used as a fluxmeter or magnetometer if implemented in circuit QED platforms.

## 1. Introduction

The high susceptibility developed by critical systems [1,2] in proximity of phase transitions is a compelling resource for metrology and sensing. For example, relevant scientific and technological applications of critical systems are bubble chambers [3] and transition-edge sensors [4]. However, even when these devices have a quantum working principle, they follow a classical sensing strategy. However, it is well known that quantum properties such as squeezing and entanglement can be used to outperform any classical sensing protocol [5]. As systems in proximity of quantum phase transitions [6] are expected to have a highly nonclassical behavior, it is natural to analyze the critical systems with a quantum-metrology perspective. In the last decade, various theoretical works have introduced different protocols able to leverage quantum critical phase transitions to achieve a fundamental advantage over classical sensing strategies [7,8,9,10,11,12,13]. However, an often-neglected fundamental hindrance limits the performances of critical quantum sensors: The diverging susceptibility is counterbalanced by the critical slowing down, which implies an extremely long protocol duration time. Only very recently it has been shown that, counterintuitively, even in the presence of the critical slowing down, the optimal limit of precision can be achieved [14]. Indeed, under standard assumptions, critical protocols can achieve the Heisenberg scaling—a quadratic growth of parameter-estimation precision—both with respect to the number of probes and with respect to the measurement time. Furthermore, a recent theoretical work [15] demonstrated that the optimal limits of precision can be achieved using finite-component phase transitions [16,17,18,19,20,21,22,23,24,25,26,27,28], which are criticalities that take place in quantum optical systems where the thermodynamic limit is replaced by a scaling of the system parameters [20,29,30,31,32,33,34]. Critical quantum sensors can then also be implemented with controllable small-scale quantum devices, without requiring the control of complex many-body systems. These results have prompted an intense research effort dedicated to designing efficient protocols [35,36,37,38,39,40,41,42,43,44,45,46,47,48,49,50,51] in terms of high estimation precision and limited measurement time, and which can be implemented with experimentally feasible operations. Practical applications in quantum magnetometry and superconducting-qubit readout were also been proposed [52].

Critical quantum metrology protocols can be divided into two main approaches. The *static* approach [7,8,9,10,11,14,15,42,43,44,45,46,47,48,53] consists of bringing the system in an equilibrium state that depends on an external perturbation (such as a magnetic field). Such equilibrium states can be represented by the ground state reached during an adiabatic sweep, or by the steady-state achieved after a long-time evolution in a driven-dissipative setting. When the system is brought in proximity of the phase transition, one can obtain a very precise estimate of the parameter by measuring an observable on the equilibrium state. In contrast, the *dynamical* approach [12,13,36] consists of preparing the probe in a known state to then apply the perturbation and monitor the system time evolution, which can also have a critical dependence on the system parameters. Recent results obtained with spin systems and finite-component transitions suggest [14] that the dynamical and equilibrium approaches have a similar scaling of the estimation precision in the thermodynamic (or parameter-scaling) limit. However, the dynamical approach can achieve a constant factor advantage over static protocols [36], and it can allow super-Heisenberg scaling in collective light–matter interaction models [51]. For fully connected models, it has recently been shown that a continuous connection [54] can be drawn between the static and dynamical approaches, identifying universal time-scaling regimes.

In the design of critical quantum sensing protocols, a variety of physical models were considered, such as many-body spin systems [14], the ensemble of emitters coupled to cavity modes [10], single atom-cavity models [15,36], and nonlinear quantum resonators [52]. To date, except for a few exceptions, most studies have focused on the parameter regime defined by thermodynamic or parameter-scaling limits, where an effective analytical description can be derived. When considering finite-component phase transition, the most widely studied case is the quantum Rabi model (QRM) [55,56,57], composed of a two-level atom coupled to a single quantum harmonic mode. This model undergoes a second-order critical phase transition in the slow-resonator limit [16,17,18,19,20,26,27,28], where the frequency of the mode and the coupling strength are sent to zero with a given scaling law. Focusing on the scaling limit, one can obtain interesting results on the growth of the estimation precision in terms of fundamental resources such as the size of probe systems or photon number, however, to assess the actual precision of practical protocols, finite values of the parameters must be considered.

In this work, we propose quantum critical sensing protocols based on a generalization of the quantum Rabi model which includes a nonlinear (two-photon) coupling term and a transversal spin bias. The linear and nonlinear interactions lead to a ground state whose dependence on the linear coupling is much stronger at the critical value, entailing the equivalent of a first-order quantum phase transition. We consider the static approach where an adiabatic sweep is used to bring the system in proximity of criticality and we perform a numerical analysis which is not limited to the scaling regime. We show that adding the nonlinear coupling and the bias improves the protocol efficiency in different ways: (1) higher estimation precision, as measured by an increase in the quantum Fisher information; (2) faster adiabatic sweep and so shorter protocol duration time, due to the larger energy gap for finite values of physical parameters; (3) an extended range of the efficient sensing region, as the position of the critical point can be tuned in the space of parameters; (4) a less challenging requirement on the implementation of the slow-resonator limit. The considered model can be feasibly implemented with atomic [58,59,60,61] and solid-state [62,63,64] quantum devices with currently available technology.

## 2. Model

The nonlinear QRM with bias is described by the Hamiltonian [58,65]

(1) $$\begin{array}{ccc} H & = & H_{0}+H_{t}+H_{\epsilon },\\ H_{0} & = & \omega a^{†}a+\frac{\Omega }{2}\sigma_{x}+g_{1}\sigma_{z}\left(a^{†}+a\right),\\ H_{t} & = & g_{2}\sigma_{z}\left[{\left(a^{†}\right)}^{2}+a^{2}\right],\;H_{\epsilon }=-\epsilon \sigma_{z}, \end{array}$$

where $\sigma_{x,y,z}$ are Pauli matrices and $a^{†}\left(a\right)$ creates (annihilates) a bosonic mode with frequency ω. The term proportional to Ω corresponds to tunneling between two states of the flux qubit in circuit QED implementations [62,66], or to the electronic-level splitting in trapped-ion implementations [59]. The strengths of linear and nonlinear couplings are denoted by $g_{1}$ and $g_{2}$ respectively. The bias term $H_{\epsilon }$ can be easily tuned by a bias current or by a static magnetic or electric field, depending on the implementation. In the slow-resonator limit ω→0, the model exhibits the analogue of both second-order and first-order phase transitions as the thermodynamic limit in a many-body system is simulated here through the infinitesimal level spacing [65]. At finite frequencies, the discontinuities in the parameter dependence of expectation values are rounded off but show remnants of criticality. It should be noted that the parity symmetry of the linear QRM ($H_{0}$) is broken by $H_{t}$ and $H_{\epsilon }$. Notice that we are considering a Hamiltonian model, neglecting the effects of decoherence and dissipation. This allows us to focus on the role played by the two-photon coupling and bias terms, with respect to the standard quantum Rabi model. In practice, this implies that the metrological performance analyzed here is strictly valid within the coherence time of potential experimental implementations. In a recent experiment [67] with circuit-QED devices, it was shown that Schrödinger cat states can be generated using an implementation of the quantum Rabi model operated at criticality. The generation of these highly nonclassical states shows that the purely quantum dynamics generated by critical systems can indeed be applied in quantum-information tasks.

## 3. Relation between Transition Order and Accuracy

To discuss the difference between the linear ($g_{2}=0$) and nonlinear ($g_{2}\neq 0$) cases for quantum metrology, let us start with zero bias ϵ=0. The model has a phase transition in the low-frequency limit ω→0 at the critical point $g_{1}=\left|g_{1c}\right|=\left|g_{s}\right|\sqrt{1-g_{2}^{2}/g_{t}^{2}}$ [65,68] with $g_{s}=\sqrt{\omega \Omega }/2$ [17,19], and $g_{t}=\omega /2$ is the critical value of $g_{2}$ beyond which the Hamiltonian (1) is no longer self-adjoined and becomes unphysical [59,69,70,71,72,73]. The transition in this limit is second-order-like at $g_{2}=0$ [17,18,19,20,26,27,28] and first-order-like at finite $g_{2}$ [65,68]. The precision (signal-to-noise ratio) of any experimental estimation of one of the parameters λ in (1) is bound by $I_{\lambda }^{1/2}$ [74], where $I_{\lambda }$ is the quantum Fisher information [14,74,75], which takes the following form for pure states

(2) $$I_{\lambda }(|\psi \rangle )=4\left(\langle \psi^{\prime }\left(\lambda \right)|\psi^{\prime }\left(\lambda \right)\rangle -{\left|\langle \psi^{\prime }\left(\lambda \right)|\psi \left(\lambda \right)\rangle \right|}^{2}\right),$$

where ${}^{\prime }$ denotes the derivative of the ground state (GS) |ψ(λ)〉 of *H* in (1) with respect to λ. Obviously, a higher QFI means a higher measurement precision.

**Figure 1:**
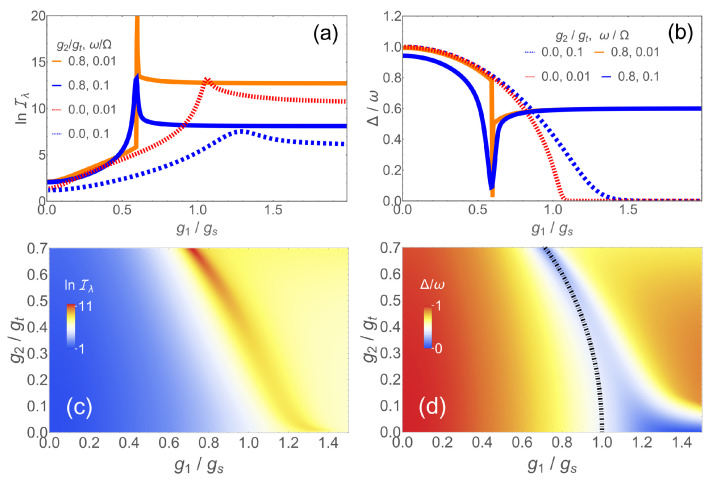
(**a**) $\ln I_{\lambda }$ for $\lambda =g_{1}/\Omega$ and different $g_{2}$: $g_{2}=0$ with ω/Ω=0.01 (dotted red line) and ω/Ω=0.01 (dashed blue line). $g_{2}/g_{t}=0.8$ with ω/Ω=0.01 (solid orange line) and ω/Ω=0.01 (solid blue line); (**b**) gap Δ/ω for the same parameters as in (**a**); (**c**) $\ln I_{\lambda }$ in the $g_{1}$/$g_{2}$ plane for ω/Ω=0.1; and (**d**) gap Δ/ω for the same parameters as in (**c**). The dashed-dotted line represents the phase boundary given in (4).

The Hamiltonian *H* has several parameters that can drive a phase transition. Let us begin with the linear coupling $g_{1}$ and set $\lambda =g_{1}/\Omega$ with Ω fixed. λ and $I_{\lambda }$ are thus dimensionless. In Figure 1a, we compare the QFI for first- and second-order scenarios, as calculated with exact diagonalization [65] by plotting $\ln I_{\lambda }$. The dashed lines illustrate the second-order case $g_{2}=0$ for two different ω. One sees that the variation of the ground state with λ and therefore the maximal value of $I_{\lambda }$ becomes larger for smaller frequencies. The QFI for comparatively large ω=0.1Ω shows a broad peak shifted away from the critical point $g_{s}$ for ω=0 due to the finite GS extension at finite frequency [19]. The peak becomes sharper at lower frequency and tends to diverge in the limit ω→0, as indicated by the dotted red line with ω=0.01 Ω. At a finite frequency, the QFI does not diverge for $g_{2}=0$. The situation changes profoundly for non-zero $g_{2}$. The GS wave function behaves much more singularly even for ω=0.1 Ω (blue solid line), leading to a narrow peak in $\ln I_{\lambda }$. Naturally, the maximal QFI is even higher for smaller frequency. By comparing the solid blue and dashed orange lines, we see that the same measurement precision can be obtained if $g_{2}\neq 0$ as in the model with $g_{2}=0$, although the mode frequency is an order of magnitude larger. Obviously, the presence of the nonlinear term in *H* simplifies the requirement to implement the slow-resonator limit.

These features of the QFI can be understood by comparing the behavior of the gap Δ between GS and the first excited state when tuning through the phase transition, shown in Figure 1b. For $g_{2}=0$, the scaled gap Δ/ω goes to zero for $g_{1}≳g_{s}$. The transition becomes continuous with Δ/ω=0 for $g_{1}\geq g_{s}$ in the limit ω→0, typical for a second-order transition. Likewise, the GS wave function changes smoothly close to $g_{s}$ leading to the lower values for the QFI. The closing of the gap means that the dynamical time scale $\Delta^{-1}$ diverges in approaching the critical coupling which means that an adiabatic sweep through $g_{s}$ would be extremely slow for ω≈0. This problem will be addressed in the next section.

On the other hand, the gap stays always finite for $g_{2}\neq 0$ due to the broken parity symmetry [65], although it changes very fast close to the critical point, even for a large ω, and therefore resembles a first-order transition. This explains the higher QFI in the nonlinear case. The QFI and the gap as a function of $g_{1}$ and $g_{2}$ are shown in the colorplots of Figure 1c,d for ω=0.1 Ω. A larger $g_{2}$ means a higher maximal QFI, which dramatically increases if $g_{2}$ reaches ∼0.6$g_{s}$. In Figure 1c, we only plot up to $g_{2}=0.7g_{t}$ as the maximal QFI for a larger $g_{2}\approx g_{t}$ would be out of scale. For these values, the system is close to the point of spectral collapse [59,71,72,73] where part of the discrete spectrum becomes continuous. Although this regime may not be easily realizable, we see that it has by far the greatest potential with regard to quantum metrology.

## 4. Preparation Time

The protocol we consider is composed of three steps: first, the system is initialized in its ground state in a region of parameters far from criticality, e.g., $g_{1}=0$, $g_{2}<g_{t}$; then, an adiabatic sweep is performed in order to bring the system in proximity of criticality; finally, a measurement is performed on the system final state. Notice that the ground state is always nondegenerate, even for vanishing values of ω, i.e., the energy gap is always finite outside the critical region, so the initial and final states are adiabatically connected on any line in the $g_{1}/g_{2}$-plane which does not cross the critical points. To estimate the time needed to perform the adiabatic sweep from λ=0 to the intended sensing value $\lambda_{s}$, we may use the condition dλ/dt≪Δ(λ) where Δ(λ) is the energy gap between the ground state and first excited state (see the supplemental material in [15]). This condition gives us an intrinsic lower bound to the time required to adiabatically prepare the system ground state for a given value of λ, even when using an adaptive sweep whose speed of variation is adjusted to the instantaneous value of the energy gap. In this way, we obtain a lower bound for the preparation time

(3) $$T\left(\lambda_{s}\right)\geq \int_{0}^{\lambda_{s}}\frac{1}{\Delta (\lambda )}d\lambda .$$

**Figure 2:**
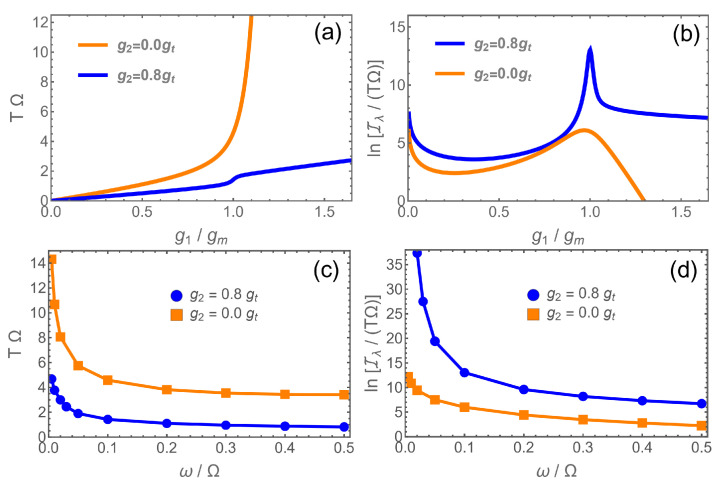
(**a**) Time *T* needed to prepare the ground state of the system at $g_{1}$ for different $g_{2}$: $g_{2}=0$ (orange) and $g_{2}=0.8g_{t}$ (blue), for ω=0.1Ω. $g_{m}$ denotes the coupling $g_{1}$ with maximal Fisher information $I_{\lambda }$. (**b**) $\ln(I_{\lambda }/\left(T\Omega \right))$ for $g_{2}=0$ (orange) and $g_{2}=0.8g_{t}$ (blue), for the same parameters as in (**a**). (**c**) Dependence of *T* at $g_{m}$ on the mode frequency ω for the two values for $g_{2}$ shown in (**a**,**b**). (**d**) $\ln(I_{\lambda }/\left(T\Omega \right))$ at $g_{m}$ as a function of ω for the same parameters as in (**c**).

In our present case, we have $\lambda =g_{1}/\Omega$. In Figure 2, we compare the preparation times for pure linear coupling $g_{2}=0$ (second-order transition) and nonlinear coupling (first-order transition) at the experimentally feasible frequency ratio ω/Ω=0.1. While the preparation time seems to diverge at the critical point (which is also the point of maximal QFI) due to critical slowing down in the first case, it stays low in the second. In Figure 2b, we plot the logarithm of $I_{\lambda }/\left(T\Omega \right)$, a figure of merit to assess the practicability of the sensing protocol. Around the coupling with maximal accuracy, $g_{m}$, the system with nonlinear coupling exhibits a precision several orders of magnitude higher than the linear one. In Figure 2c,d, *T* and $\ln(I_{\lambda }/\left(T\Omega \right))$ taken at $g_{m}$ are shown as a function of ω/Ω. The preparation time rises if one approaches the low frequency limit for linear and nonlinear coupling alike because the phase transition features become more pronounced and the gap in the critical region shrinks. Nonetheless, one can see that the preparation time in the presence of nonlinear coupling is much lower than without it. For values above ω/Ω∼0.2, the time does not change much in both cases. Likewise, the “effective accuracy” as measured by $\ln(I_{\lambda }/\left(T\Omega \right))$ slowly drops for larger values of ω, while the nonlinear system keeps a much higher precision.

## 5. Behavior of the Wave Function

**Figure 3:**
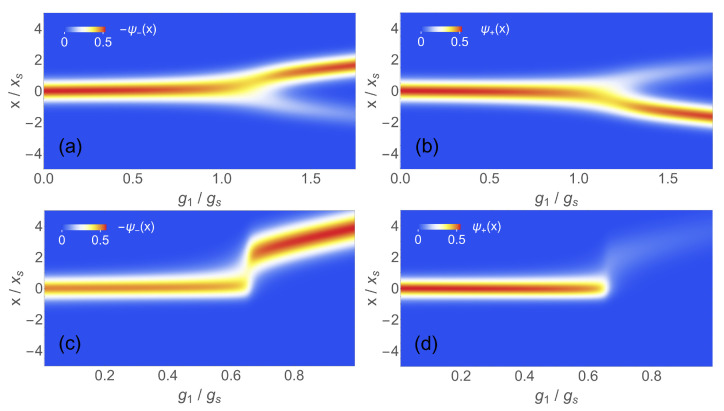
Ground state wave function $\psi_{\pm }\left(x\right)$ for spin component + or − as a function of $g_{1}$ at ω/Ω=0.1: (**a**) $-\psi_{-}\left(x\right)$ for $g_{2}=0$; (**b**) $\psi_{+}\left(x\right)$ for $g_{2}=0$; (**c**) $\psi_{-}\left(x\right)$ for $g_{2}=0.75g_{t}$; and (**d**) $\psi_{+}\left(x\right)$ for $g_{2}=0.75g_{t}$. Here, $x_{s}=\sqrt{2}g_{s}/\omega$.

As mentioned above, the high sensitivity of quantum metrology results from the sudden change of the GS wave function |ψ〉 in the vicinity of the critical point. In Figure 3a,b, we show the components of $|\psi \rangle ={\left(\psi_{+}\left(x\right),\psi_{-}\left(x\right)\right)}^{T}$ in position space for $g_{2}=0$ and as a function of $g_{1}$. The frequency ω is relatively large (ω/Ω=0.1) so that the transition is smeared out. Below $g_{1}\approx g_{s}$, both spin components of |ψ〉 are centered around x=0 which corresponds to unbroken left/right-symmetry. Around $g_{s}$, the upper component is displaced to the left and the lower component to the right. This does not mean that the parity symmetry of the model with the Hamiltonian $H_{0}$ is broken for $g_{1}>g_{s}$, because the parity operator $e^{i\pi a^{†}a}\sigma_{x}$ acts in both spin and position space. Nevertheless, the change in the GS wave function in position space is the analogue of a symmetry breaking quantum phase transition in the QRM. For vanishing nonlinear coupling $g_{2}$, the change in both components is smooth, as seen in Figure 3a,b. The situation is quite different if the nonlinear coupling is turned on: for non-zero $g_{2}$, which breaks the parity symmetry of $H_{0}$, we essentially have the same behavior of $\psi_{\pm }\left(x\right)$ for $g_{1}<0.66g_{s}$ as in the linear case. However, at $g_{1}\approx 0.66g_{s}$, the wave functions change abruptly: basically, the whole weight is transferred to the right and lower branch $\psi_{-}\left(x\right)$ and the parity symmetry is strongly broken. Of course, this is no symmetry breaking in the usual sense because parity is already broken on the Hamiltonian level. The fast change of |ψ(x)〉 in tuning through the transition region is responsible for the large QFI, while the gap to the first excited state always remains non-zero.

## 6. Extended Range Quantum Sensing

Up to now, we set the bias ϵ to zero to demonstrate the main differences between the linear and nonlinear models with regard to quantum sensing. From Figure 1c,d, we see that, in varying $g_{2}$ between zero and $0.7g_{t}$, we can drive the critical coupling $g_{1}$ which is the quantity to be measured, from $g_{s}$ to lower values ∼0.7$g_{s}$, thus extending the range of couplings which can be measured with an accuracy enhanced by criticality. A much larger region of couplings becomes available if the bias ϵ is varied as well which can be easily achieved, e.g., in circuit QED platforms.

**Figure 4:**
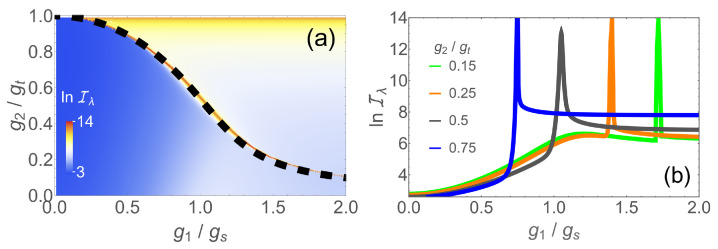
(**a**) $\ln I_{\lambda }$ in the $g_{1}$/$g_{2}$ plane at a fixed frequency ω/Ω=0.1 and finite bias ϵ=0.1Ω. The black dashed line denotes the analytic phase boundary where $I_{\lambda }$ is maximal; (**b**) $\ln I_{\lambda }$ as a function of $g_{1}$ for different nonlinear couplings: $g_{2}/g_{t}=0.75$ (blue), 0.5 (dark gray), 0.25 (orange), 0.15 (green).

In Figure 4a, we show the QFI in the $g_{1}$/$g_{2}$ plane in the presence of finite bias ϵ=0.1Ω at ω=0.1Ω. The phase transition occurs along the thin red line indicating the sharp maximum of the QFI. The transition line is accurately given by a semi-classical calculation (black dashed line) in closed form as [65]

(4) $$\begin{array}{cc} g_{1c}^{\epsilon } & =g_{s}\left[1+\frac{g_{t}\epsilon }{g_{2}\Omega }\right]\sqrt{1-g_{2}^{2}/g_{t}^{2}}, \end{array}$$

(5) $$\begin{array}{cc} \epsilon_{c} & =\frac{g_{2}}{g_{t}}\left[\frac{g_{1}}{g_{s}\sqrt{1-g_{2}^{2}/g_{t}^{2}}}-1\right]\Omega . \end{array}$$

This phase boundary no longer cuts the *x* axis at a finite value of $g_{1}$ as in Figure 1c, but allows for arbitrary large critical values of $g_{1}$ for non-zero $g_{2}$. The range of accessible couplings is therefore also extended to values above $g_{s}$. As such, the whole range $0<g_{1}<\infty$ can be measured with enhanced precision if the couplings $g_{2}$ and ϵ are properly tuned.

The QFI has a peak along the phase boundary. This is shown in Figure 4b for various values of $g_{2}$. One may notice a shallow maximum of the lines for $g_{2}/g_{t}=0.15$ and 0.25, around $g_{1}/g_{s}=1.2$ before the sharp peak associated with the first order transition. This originates in a second-order transition because the system is located in the vicinity of a tricritical point [65]. However, these maxima only lead to the marginal enhancement of QFI and play no role in the optimal measurement protocol. The contrast between the shallow maximum and the sharp peak for the same $g_{2}$ again demonstrates the much higher measurement accuracy made possible by a first-order-like transition compared to a second-order transition.

## 7. Magnetometry

**Figure 5:**
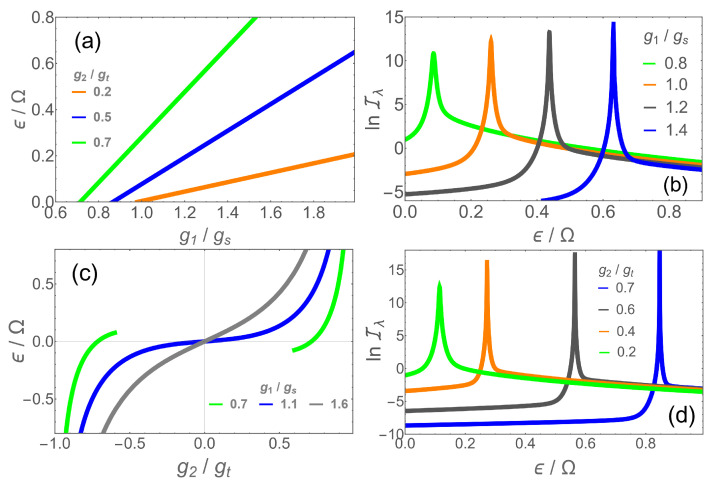
(**a**) Phase boundaries as a function of $g_{1}$ at $g_{2}/g_{t}=0.2$ (orange), 0.5 (blue), 0.7 (green); (**b**) $\ln I_{\lambda }$ for λ=ϵ/Ω as a function of ϵ for $g_{2}/g_{t}=0.7$ and $\left(g_{1}/g_{s},\omega /\Omega \right)=\left(0.8,0.1\right)$ (green), (1.0,0.2) (orange), (1.2,0.3) (dark gray) and (1.4,0.4) (blue). (**c**) Phase boundaries as a function of $g_{2}$ at $g_{1}/g_{s}=0.7$ (green), 1.1 (blue) and 1.6 (gray). (**d**) $I_{\lambda }$ as function of ϵ for $g_{1}/g_{s}=0.7$ at $\left(g_{2}/g_{t},\omega /\Omega \right)=\left(0.2,0.2\right)$ (green), (0.4,0.2) (orange), (0.6,0.3) (dark gray) and (0.7,0.4) (blue).

The general Hamiltonian (1) contains five parameters, all of which can be subjected to quantum metrology. We focused, as an example, to the linear coupling $g_{1}$ but other parameters are also interesting from a metrological point of view. The bias ϵ is of particular interest as it can be directly proportional to the intensity of external electric or magnetic fields in atomic and circuit–QED implementations, respectively. Using such a platform, it would be possible to construct a magnetometer analogous to a SQUID with enhanced precision. We computed the QFI for λ=ϵ/Ω in different parameter regions at finite frequencies. The results are shown in Figure 5b,d as a function of the measured quantity ϵ for non-zero values of $g_{2}$ to take advantage of the nonlinear coupling. Qualitatively, we find the same features as for the previous case with $\lambda =g_{1}/\Omega$. In Figure 5a,c, the phase boundaries are shown in the ϵ/$g_{1}$ plane and the ϵ/$g_{2}$ plane, respectively. In each case, the whole range for ϵ can be attained by a phase boundary point if $g_{1}$ and $g_{2}$ are adjusted through a suitable adiabatic preparation process.

## 8. Discussion

Via a study of the QFI and the gap of the nonlinear quantum Rabi model with bias, we compared the critical metrology provided by quantum phase transitions of a different order. While the model with only linear coupling shows a transition of second-order type with a closing gap and smooth GS wave function, the transition of the model with additional nonlinear coupling can be classified as first order and featuring a finite gap and a discontinuous change of the GS wave function. The reason for this difference is that the broken parity symmetry of the nonlinear model which manifests itself in the GS wave function only at and above the critical point. In contrast, the linear, parity symmetric model has a GS changing smoothly across the transition. This leads to a dramatic increase in the QFI close to criticality in the nonlinear case. Moreover, the critical slowing down due to the gap closing which extends the preparation time in the linear model is absent for nonlinear coupling. A third advantage of the nonlinear over the linear model is the possibility of avoiding the slow-resonator limit as frequency rations of ω/Ω∼0.1 are sufficient to utilize the critical quantum enhancement of the measurement precision. This condition substantially eases the requirements for an experimental implementation. Finally, adding a standard bias term to the Hamiltonian extends the measurement range for the couplings to all realizable values because the critical point can be shifted by adjusting the bias. On the other hand, one may construct a new type of magnetometer with critically enhanced precision if the bias itself is subjected to the measurement.

Therefore, the extension of the standard quantum Rabi model by including a nonlinear coupling and the bias term may lead to a major improvement of quantum metrology in not just one but several respects. 

## Data Availability

Not applicable.
